# 1661. Evaluation of Cefazolin < 3 g vs. 3 g Treatment Doses for Cellulitis in Patients Who Weigh ≥ 120 kg within a Large, Community Health System

**DOI:** 10.1093/ofid/ofac492.127

**Published:** 2022-12-15

**Authors:** Krista Gens, Michael Wankum, Elizabeth B Hirsch, Brandon Gagnon

**Affiliations:** Abbott Northwestern Hospital, Minneapolis, Minnesota; Abbott Northwestern Hospital, Minneapolis, Minnesota; University of Minnesota College of Pharmacy, Minneapolis, MN; Abbott Northwestern Hospital, Minneapolis, Minnesota

## Abstract

**Background:**

Cefazolin 3 g is recommended for obese patients weighing > 120 kg preoperatively; however, there is no available evidence to suggest 3 g for treatment dosing of cefazolin in this population. This study aims to provide the first clinical data of its kind on the efficacy and safety of high-dose cefazolin in the treatment of cellulitis in obese patients using a modified Desirability of Outcomes Ranking (DOOR) methodology.

**Methods:**

This is a multi-center, retrospective cohort study including adults weighing > 120 kg at the time of admission who received ≥ 48 hours of cefazolin monotherapy for cellulitis. Patients were gathered in a 3:1 ratio between the < 3 g dosing (standard-dose; SD) and 3 g dosing (high-dose; HD) groups. Patients were excluded if they had co-infections, bacteremia, bilateral lower extremity cellulitis, or if they received > 48 hours of concomitant antibiotics. The primary endpoint of efficacy and safety as determined by modified DOOR criteria was compared between obese patients who received HD and those who received SD between 1/1/2021 and 12/31/2021. The DOOR is a patient-centered methodology that uses combined efficacy and safety endpoints to help inform better clinical decision-making.

**Results:**

A total of 68 patients were included; 51 received SD and 17 received HD cefazolin. There were no differences in mean age nor baseline kidney function or severity of illness between groups. The median patient weights were 140.6 kg and 160 kg for the SD and HD groups, respectively (p = 0.07). Patients achieving clinical success (resolution of cellulitis within 10 days or clinical improvement upon discharge) was higher in the HD vs SD group (70.6% vs 41%; p=0.036) (Figure 1).

**Conclusion:**

Cefazolin 3 g was associated with higher clinical success in patients > 120 kg with cellulitis. Further study is necessary to confirm these results.

**Disclosures:**

**Elizabeth B. Hirsch, PharmD, FCCP, FIDSA**, Melinta: Advisor/Consultant|MeMed: Advisor/Consultant|Merck: Advisor/Consultant|Merck: Grant/Research Support.

**Figure 1.**
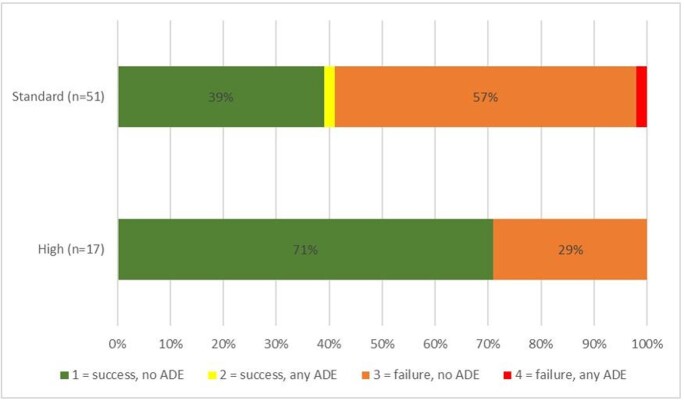
Desirability of outcome ranking distribution for cefazolin standard dosing vs high dosing

